# Expression of recombinant Araraquara Hantavirus nucleoprotein in insect cells and its use as an antigen for immunodetection compared to the same antigen expressed in *Escherichia coli*

**DOI:** 10.1186/1743-422X-8-218

**Published:** 2011-05-11

**Authors:** Alex M Machado, Aline RSR Machado, Marcos L Moreli, Bergmann M Ribeiro, Luiz TM Figueiredo, Jose LC Wolff

**Affiliations:** 1 Centro de Pesquisa em Virologia, Faculdade de Medicina de Ribeirão Preto, Universidade de São Paulo, Av. Bandeirantes 3900, Monte Alegre, Ribeirão Preto-SP,14090-900, Brazil; 2 Universidade Federal de Goiás, Rodovia BR364, Km 192, Parque Industrial, Jataí-GO, 75800-000, Brazil; 3 Laboratório de Microscopia Eletrônica e Virologia, Departamento de Biologia Celular, Instituto de Ciências Biológicas, Universidade de Brasilia, Campus Universitário Darcy Ribeiro, Brasília, 70910-900, Brazil; 4 Centro de Ciências Biológicas e da Saúde, Universidade Presbiteriana Mackenzie, Rua: Consolação 930, Consolação, São Paulo-SP, 01302-090, Brazil

## Abstract

**Background:**

Antigens for Hantavirus serological tests have been produced using DNA recombinant technology for more than twenty years. Several different strategies have been used for that purpose. All of them avoid the risks and difficulties involved in multiplying Hantavirus in the laboratory. In Brazil, the Araraquara virus is one of the main causes of Hantavirus Cardio-Pulmonary Syndrome (HCPS).

**Methods:**

In this investigation, we report the expression of the N protein of the Araraquara Hantavirus in a Baculovirus Expression System, the use of this protein in IgM and IgG ELISA and comparison with the same antigen generated in *E. coli*.

**Results:**

The protein obtained, and purified in a nickel column, was effectively recognized by antibodies from confirmed HCPS patients. Comparison of the baculovirus generated antigen with the N protein produced in *E. coli *showed that both were equally effective in terms of sensitivity and specificity.

**Conclusions:**

Our results therefore indicate that either of these proteins can be used in serological tests in Brazil.

## Background

The genus Hantavirus of the family *Bunyaviridae *includes more than 30 viral species distributed throughout the world. These rodent-borne viruses have been increasing importance in global public health, being transmitted to humans through contact with contaminated feces or intimate contact with infected rodents [1].

Hantaviruses have diameters ranging from 71 to 149 nm (average diameter: 112 nm). The virus particle is enveloped by a lipid bilayer of approximately 7 nm thick in which its surface glycoproteins (Gn and Gc) are attached. The nucleocapsids are formed by a delicate web of filamentous granular protein (N), which protects and interacts with each of the 3 segments of viral RNA (vRNA). The RNA molecules that form the virus genome are single stranded with negative polarity. They have complementary sequences at the 3' and 5' end, which allows the viral RNA remain circular within the virion. The RNA segments are named according to their size as L (large), M (medium) and S (small). The L segment has approximately 6500 nucleotides and encodes the viral RNA-dependent RNA polymerase. This enzyme is responsible for transcription and replication of the viral genome and, as expected for RNA virus with negative polarity, is present in the virion. The M segment (medium), with 3600 to 3800 nucleotides, encodes a polyprotein precursor (GPC) which, after cleavage, leads the two viral surface glycoproteins, Gn and Gc. The S segment (small), with 1300 to 2100 nucleotides, encodes the N protein [2]. This protein is abundantly produced after infection and is responsible for several important viral functions such as, preventing the degradation of the vRNA and interacting with other proteins at the end of the infection process favoring viral assembly [3]. The protein also induces strong humoral response, in both, patients and rodents, with antibodies directed to the 3 major epitopes of N, which are located in the amino terminal protein region [4].

The human infection by Hantavirus can cause 2 diseases depending on the region of the globe where the patient became infected: hemorrhagic fever with renal syndrome (HFRS) in Asia and Europe and the Cardio-pulmonary Syndrome (HCPS) in the Americas. While the former is transmitted by *Murinae *and *Arvicolinae *rodents of the Old World, the latter is transmitted by *Sigmodontinae *rodents of the New World [1].

HCPS was reported for first time in the Americas, in 1993, causing pneumonia with respiratory failure among Navajo Indians in the Four Corners region of USA. In the same year, the first cases were reported in the Brazilian city of Juquitiba and a couple thousand of HCPS cases have been reported across America [5,6]. According to the Ministry of Health of Brazil, by the year 2009, about 1200 HCPS cases were reported, with a 39% case fatality rate [7]. At least 5 Hantavirus are the causatives of HCPS in Brazil: Juquitiba virus (JUQV), Araraquara virus (ARAV), Laguna Negra virus (LNV), Castelo dos Sonhos virus (CASV) and Anajatuba virus (ANAJV). Among these, ARAV has been the main causative of HCPS, occurring in the "*Cerrado" *landscapes of Southeast and Central Plateau and producing about 49% of fatalities [8]. In fact, ARAV has been considered the most virulent Hantavirus in Brazil and probably in the world [8].

The diagnosis of HCPS in Brazil is based on the clinical presentation previous contact with rodents and detection of IgM antibodies to Hantavírus [9]. In Brazil, until a few years ago the ELISA for Hantavirus diagnosis was performed only by public health laboratories, using recombinant proteins of Sin Nombre virus (SNV) and Andes virus (ANDV), from the Centers for Disease Control (USA) and Instituto Carlos Malbran (Argentina), respectively, as antigens [1].

Recently, a recombinant N protein of ARAV was produced [10]. The RNA used for the synthesis of the vector was obtained from virus particle taken from blood samples of an HCPS patient. The entire S segment of the virus was amplified and sequenced. The analysis of the sequence revealed a segment of 1858 nucleotides with an open reading frame that encodes a protein of 429 amino acids. The nucleotide sequence confirmed a high identity with the N protein gene of ARAV. The entire gene was cloned in the vector pET200D and the N protein was expressed in *Escherichia coli *BL21 strain [10]. The expression of the recombinant protein was confirmed by the detection of a 52-kDa protein by Western blot using a pool of human sera obtained from HCPS patients [10]. This recombinant protein was purified and has been used as antigen in ELISA for detection of IgG and IgM antibodies against Hantavirus [10]. However, studies comparing serological methods for detection of Hantavirus, indicate that recombinant proteins expressed in baculovirus expression system using insect cells, may be more suitable, in terms of expression levels and also easier for purification [11-13].

Here we report the expression of the N recombinant protein of ARAV in insect cells using the baculovirus expression system, the use of this protein as antigen in ELISA was evaluated and compared to antigen produced in *Escherichia coli*.

## Methods

### Cloning of Nucleoprotein gene in the baculovirus transfer vector

The N gene of Araraquara Hantavirus used in this study was obtained from the plasmid pET-N-ARA [10]. This gene was originally isolated by RT-PCR from the serum of patients with HCPS and cloned into the expression vector pET Directional TOPO^® ^(Invitrogen USA) [10]. The DNA from this plasmid was used as template for Polimerase Chain Reaction (PCR) using the Forward Histidine and Reverse Nucleoprotein primers (Table 1). The amplicon containing the entire N gene was purified using the GFX purification kit (Amersham, USA) and cloned into the vector PCR2.1 (Invitrogen USA), following manufacturer's instructions. The plasmid obtained, named PCR 2.1-Hist-N, was subsequently digested with *BglII *(Biolabs USA) and *XmaI *(Biolabs USA) restriction enzymes in order to release the fragment of interest. After purification, this fragment was inserted into the transfer vector pSynXIV VI^+^X3 [14]. This transfer vector contains two promoters (pSYN and pXIV) in tanden driving the expresson of the foreign gene. The transfer vector containing the insert and named pSyn-Hist-N was sequenced in order to confirm the correct insertion. The sequencing reaction was carried out using the BigDye v.3 kit (Applied Biosystems USA) using specific primers (Table 1) and run in an ABI Prism 377 sequencer (Applied Biosystems USA). The sequences obtained were assembled using the CodonCode Aligner program version 1.3.4 (CodonCode Corporation). Subsequently, these sequences were analyzed using the ORF finder and BLAST software (*National Center for Biotechnology Information *- NCBI).

**Table 1:** Specific primers for amplification/sequencing of Araraquara hantavirus nucleoprotein gene and amplification of betagalactosidase gene.

*Primers* *name*	*Primer* *Sequence*	*Function*
For. His.	5' GAAGATCTATGCGGGGTTCTCATCAT 3'	Amplification of N gene.
Rev. NP.	5 TAACCCGGGTCACAGCTTTAAGGGTCC 3'	Amplification of N gene.
Int. NP.	5' AGACAGCAGACTGGAAG 3'	Sequencing of N gene.
For. pSyn	5' GGGCCAAGCTTGGCGTTATTG 3'	Sequencing of N gene
Rev. pSyn	5' TCTGTAAATCAACAACGCACAG 3'	Sequencing of N gene
For. β-gal	5' TTCACTGGCCGTCGTTTTACAACGTCGTGA 3'	Check of Recombinant Baculovírus Purity
Rev. β-gal	5' ATGTGAGCGAGTAACAACCCGTCGGATTCT3'	Check of Recombinant Baculovírus Purity

**Primers name: For. His **- Forward Histidina; **Rev. NP **- Reverse Nucleoprotein; **Int. NP**. Intermediate Nucleoprotein; **For**. **pSyn**. Forward vector pSyn; **Rev. pSyn**. Reverse vector pSyn; **For. β-gal**. Forward gene betagalactosidase; **Rev. β-gal**. Reverse gene betagalactosidase.

### Construction of the recombinant baculovirus

Monolayers of SF-9 cells (1 × 10^6 ^cells or 50 to 70% cell density) were co-transfected with a solution containing 0.5 μg of the DNA of baculovirus vSynVI-gal linearized with *Bsu36I *and 1 μg of the recombinant plasmid, pSyn-Hist-N previously incubated with a 50 ul solution of lipofectin (Gibco-BRL). After incubation, for 3 hours, at 27°C, the cell culture medium was exchanged for fresh medium and the cell culture was further incubated at 27°C for 5 to 7 days. The vSynVI^-^gal virus is a recombinant baculovirus derived from the *Autographa californica multiple nucleopolyhedrovirus *(AcMNPV) containing the β-galactosidase gene (*lac-Z*) from *E. coli *in place of the occlusion body protein gene (*polh*) [14]. These cells were then observed under an optical light microscope, to visualize viral occlusion bodies (indicative of recombination, since the pSyn-Hist-N vector has, besides the N gene, the *polh *gene). The cell supernatants, containing recombinant viruses, were removed and stored for further confirmation of gene insertion as well as for titration and purification of the recombinant viruses. To confirm the insertion of the gene of interest, the DNA of the recombinant baculovirus was extracted and a PCR with specific primers, Forward Histidine and Reverse Nucleoprotein, (Table 1) was performed.

### Purification and titration of recombinant baculovirus

Serial dilutions of supernatant of the infected cells (5 to 7 days after transfection) were performed and used to infect Sf9 cells in 96 well plates. The identification of recombinant viruses infecting these cells was done by the presence of occlusion bodies in the nucleus. The cells infected with the highest dilutions and having occlusion bodies in the nucleus were selected and had the supernatants used for other serial dilutions. This process was done for 3 times in order to obtain purified recombinant viruses. The purity of the recombinant virus was checked by a PCR that amplifies the β-galactosidase gene, using specific primers, forward β-gal and reverse β-gal (Table 1). Knowing that this gene was replaced by the gene of interest, the absence of β-galactosidase indicates an adequate purity of the recombinant virus. The supernatant of the infected cells with the purified recombinant virus was collected, titrated by plaque assay, and stored at 4°C.

### Production and analysis of recombinant proteins

Sf9 cells (2 × 10^6 ^cells/ml) grown in 75 mm^2 ^culture flasks were infected with 5 MOI of the recombinant virus and incubated at 27°C. After 72 h p.i. the cells were collected and analyzed for production of recombinant proteins in both, native and denatured forms. Thus, 2 samples of the infected cells were used. The first sample was collected by low speed centrifugation in a microcentrifuge, washed with PBS and lysed in a buffer containing 50 mM NaH_2_PO_4_, 300 mM NaCl, 1% Tween 20, pH8.0 and protease inhibitors for 1 h at 4° C under agitation. After this period, the cell lysate was centrifuged at 10,000 × g for 10 minutes and the supernatant was stored at -70°C. The second sample was submitted to the same procedure, but with lysis buffer containing 6 M urea. Samples of both cell lysates containing the recombinant protein were analyzed by polyacrylamide gel electrophoresis (SDS-PAGE) and stained with Coomassie blue.

### Purification and antigenic analysis of recombinant proteins

The denatured protein extract was passed through a column of nickel (Ni-NTA Purification System - Qiagen, USA) which binds specifically to the histidine tail present in the recombinant protein, allowing its purification. After elution with a buffer containing: 50 mM NaH_2_PO_4_, 300 mM NaCl, 1% Tween 20, 6 M urea and protease inhibitors pH 5.6 or pH 4.2, this eluate was checked for purity by SDS-PAGE and stained with Coomassie blue. The crude extract from infected SF9 cells with recombinant baculovirus and crude extract from uninfected SF9 cells were subjected to a western blot using a Hantavirus immune serum from a HCPS convalescing patient. Anti-Human IgG linked to peroxidase (Sigma, USA) was used as secondary antibody and DAB (Sigma, USA) as substrate.

### Human sera samples

Human sera obtained from 30 HCPS suspect patients, collected from 2005 to 2009 at hospitals in the cities of Ribeirão Preto, Sertãozinho e Jardinópolis were tested by IgG ELISA using the N recombinant proteins of ARAV as antigens. Additionally, 50 sera collected from participants of a Hantavirus serologic survey in the cities of Paraiso and Belmonte, state of Santa Catarina, were also tested by ELISA.

### Enzyme Linked Immunosorbent Assay- ELISA

An ELISA using recombinant protein produced in insect cells was standardized using the protocol previously established by Figueiredo and collaborators (2008) [10]. The microplates of 96 wells were divided in two parts. The wells in the first part of the plate, including rows A to D, columns 1 to 12, were coated with 2.0 μg/ml of the N recombinant protein in carbonate-bicarbonate buffer pH 9,6. The second part of the plate, rows E to H columns 1 to 12, had the wells coated with the negative antigen control (crude extract from uninfected Sf9 cells). After an overnight incubation, at 4°C, the microplates were washed 6 times with PBST buffer (Phosphate Buffer plus 20% Tween). All tested sera (positive and negative controls) were diluted 1:100 in PBS containing 10% skimmed milk and added into duplicate wells in both parts of the microplate (the part coated with N recombinant protein and the part containing the negative antigen). The microplates were washed as described above and incubated at 37°C, for 1 h, with 50 μL of an anti-human IgG immunoglobulin - peroxidase conjugate (Sigma USA), diluted 1:2000. The microplates were washed again, as previously described, and 100 μl of 2,2 '-azin-bis (3-ethylbenzthiazoline-6-sulfonic acid) (ABTS) substrate (Sigma-Aldrich, USA) was added to the microplate wells. The test was read in a spectrophotometer at 405 nm. The cut off of the test was considered as the mean plus 3 standard deviations of the optical density (OD) values obtained from all negative control samples, after subtraction of the OD from all these negative sera in rows E to H. The IgM ELISA was also standardized using the same protocol performed for IgG ELISA using, as secondary antibody, anti-human IgM immunoglobulin - peroxidase conjugate (Sigma USA), diluted 1:2000.

### Comparison of ELISAs using recombinant N protein produced in Baculovirus and E. coli expression systems

Thirty patients suspect of HCPS were tested by the IgG ELISA previously described, coated with N recombinant proteins of ARAV produced in Baculovirus (rN-Bac) or *E. coli *(rN-*E. coli*) expression systems. OD values, as well as cut-off values of tests using both antigens were compared regarding to sensitivity and specificity. Positive samples by IgG ELISA were also tested by IgM ELISA using both N recombinant proteins of ARAV as antigens. Titles of samples and OD values, as well as cut-off values of tests using both antigens were also compared. The IgG ELISA was also used to test sera of 50 volunteers from Paraíso and Belmonte cities.

## Results

### N gene amplification and cloning

The RT-PCR amplification of the N gene of Araraquara Hantavirus generated a fragment of approximately 1287 nt. This gene was subsequently cloned into a transfer vector pSynXIVVI+X3 (5.8 Kb) producing a 7.1 Kb plasmid [14]. The nucleotide sequence of the insert (cassette pSyn-Hist-N) showed 100% identity with the S segment sequence of Araraquara virus deposited in the GenBank (EF571895 and AF3073225). The ORF contained in the insert had 1287 nt, encoding a putative protein of 429 amino acids which also shown to be identical to the N protein of Araraquara virus.

### Construction, purification and titration of the recombinant baculovirus

Five days after the co-transfection that produced the recombinant viruses, the Sf9 cells showed presence of polyhedra (viral occlusion bodies) in the cells nuclei, signaling the infection, of more than 80% of the cells. The Hantavirus N gene was also found inserted in the genome of the new vSyn-Hist-N recombinant baculovirus and it was successfully purified by serial dilution which was confirmed by the lack of amplification of the β-galactosidase gene in a PCR. The vSyn-Hist-N was obtained at a titer of 1.3 x10^7 ^pfu/ml.

### Production, purification and antigenic analysis of recombinant protein

**Figure 1:**
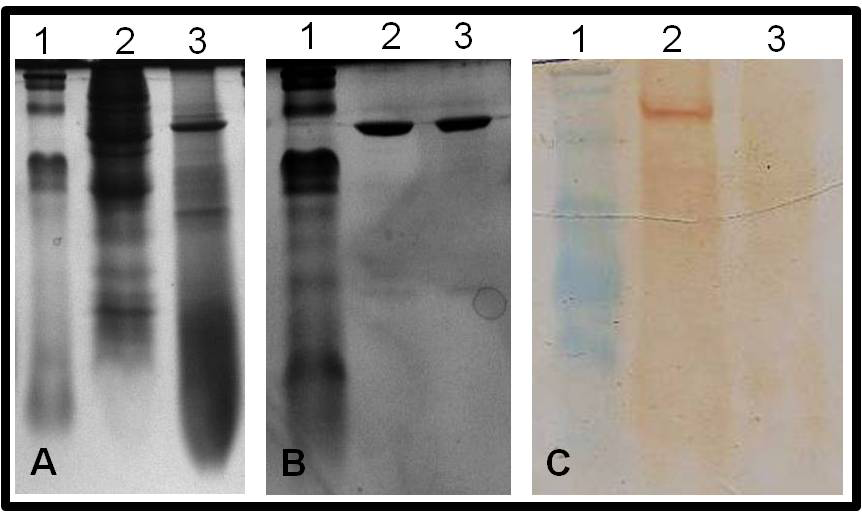
**Expression, purification and antigenic analysis by western-blot of recombinant Nucleoprotein, expressed in Baculovírus expression system**. **A**. Polyacrilamide gel electrophoresis 12,5% stained with Coomassie blue showing the expression of recombinant N protein. 1. BenchMark Protein Ladder (Bio-rad-USA), 2. Crude extract from uninfected Sf9 cells, 3. Crude extract from infected SF9 cells with recombinant Baculovirus. **B**. Polyacrilamide gel electrophoresis 12,5% stained with Coomassie blue showing the purification of recombinant N protein. 1. BenchMark Protein Ladder (Bio-rad-USA), 2/3. Purified recombinant N protein. **C**. Western-blot showing the recombinant N protein detection by HCPS serum sample. 1. BenchMark Protein Ladder (Fermentas-BRL), 2. Crude extract from infected SF9 cells with recombinant baculovirus showing the detection of recombinant N protein, 3. Crude extract from uninfected SF9 cells without detection of recombinant N protein.

The expressed Hantavirus N protein was extracted with a buffer solution containing urea. Thus, large amounts of the 52 kDa N recombinant protein were obtained after this denaturing extraction (Figure 1A). Native extraction of N recombinant protein was also investigated. However, only small amounts of recombinant N protein were obtained by this method (data no shown). The protein was purified using nickel column (Figure 1B). The crude extract from infected SF9 cells with recombinant baculovirus and crude extract from uninfected SF9 cells was analyzed by western blot. Sera from HCPS patients were able to react, in this test, with the N recombinant protein (rN-Bac) present in the extract of infected Sf9 cells, as shown in Figure 1C.

### Comparison of ELISAs using rN-Bac and rN-E. coli as antigen

Sera from 30 HCPS suspected patients were diluted 1:100 and tested by IgG-ELISAs, using rN-Bac or rN-*E. coli *as antigen. Eleven sera (37%) were positive and 19 (63%) were negative by both tests. As shown in Table 2 no significant difference (p > 0.05) in optical densities and similar cut-off values were observed in both IgG ELISAs (0.172 to rN-Bac and 0.193 to rN-*E. coli*). These eleven positive sera were later tested by Hantavirus rN-Bac and rN-*E. coli *IgM ELISA. Six sera (54,5%) were positive, showing titles between 100 and 200. The other 5 sera (45,5%) were negative by both tests (Table 3). No significant differences were observed (p > 0.05) on optical densities or serum titers. Similar cut-off values were observed in both IgM ELISAs (0.189 to rN-Bac and 0.201 to rN-*E. coli*). Hantavirus rN-Bac and rN-*E. coli *IgG-ELISAs were also used to test 50 samples of volunteers from the towns of Paraíso and Belmonte, State of Santa Catarina, showing 12 positives (24%) and 38 (76%) negatives in both tests. As we previously observed with sera from the 30 HCPS suspected patients, no significant difference (p > 0,05) in optical densities and similar cutt-off values (0.274 for rN-Bac and 0.290 for rN-*E. coli*) were observed for the 50 samples of volunteers, as shown in Table 4. Based on this comparison with the Hantavirus IgG or IgM ELISA using rN-*E. coli *as antigen, the antigen commonly used in Brazil, the rN-Bac IgG or IgM -ELISA showed 100% sensitivity, specificity and predictive values.

**Table 2:** Comparative IgG ELISA-rN-Bac and IgG ELISA-rN-*E. coli *against sera suspected HCPS received in the Virology Research Center - FMRP.

*N° Patients*	*Sex*	*Age*	*rN-Bac* *OD*	*rN-E. coli* *OD*	*Results^1^*
1	M	20	1.287	1.173	(+)/(+)
2	F	23	0.160	0.181	(-)/(-)
3	M	53	0.151	0.187	(-)/(-)
4	M	I	0.989	1.090	(+)/(+)
5	M	49	0.159	0.156	(-)/(-)
6	F	I	0.101	0.111	(-)/(-)
7	F	I	0.092	0.097	(-)/(-)
8	M	39	0.105	0.143	(-)/(-)
9	M	25	1.430	1.560	(+)/(+)
10	M	39	0.107	0.131	(-)/(-)
11	M	30	0.110	0.107	(-)/(-)
12	F	34	0.954	1.102	(+)/(+)
13	M	44	0.095	0.078	(-)/(-)
14	M	I	1.153	1.051	(+)/(+)
15	M	28	1.502	1.362	(+)/(+)
16	F	40	0.113	0.134	(-)/(-)
17	M	38	0.117	0.118	(-)/(-)
18	F	25	1.021	0.897	(+)/(+)
19	F	23	0.087	0.091	(-)/(-)
20	M	39	0.127	0.162	(-)/(-)
21	M	40	0.121	0.154	(-)/(-)
22	M	I	1.923	1.597	(+)/(+)
23	M	38	0.118	0.168	(-)/(-)
24	M	I	1.720	1.614	(+)/(+)
25	M	I	0.133	0.147	(-)/(-)
26	F	15	1.133	1.315	(+)/(+)
27	F	22	0.108	0.133	(-)/(-)
28	M	52	0.159	0.178	(-)/(-)
29	M	I	1.081	1.209	(+)/(+)
30	M	46	0.136	0.133	(-)/(-)

**OD **- Optical Density. **ELISA - **Enzyme Linked immunosorbent assay. **Bac - **Baculovirus. ***E. coli *- ***Escherichia coli*. 1. Result of assay used IgG-ELISA-rN-Bac and IgG-ELISA-rN-*E. coli*.

**Table 3:** Comparative IgM ELISA-rN-Bac and IgM ELISA-rN-*E. coli *against confirmed sera of HCPS received in the Virology Research Center - FMRP.

		*rN-Bac*		*rN - E. coli*		
			
*N° Patients*	*Genus*	*OD*	*Title Sample*	*OD*	*Title Sample*	*Results^1^*
1	M	0.142	No reagent	0.158	No reagent	(-)/(-)
2	F	0.324	1:100	0.295	1:100	(+)/(+)
3	M	0.127	No reagent	0.139	No reagent	(-)/(-)
4	M	0.339	1:200	0.351	1:200	(+)/(+)
5	M	0.114	No reagent	0.097	No reagent	(-)/(-)
6	F	0.358	1:200	0.327	1:100	(+)/(+)
7	F	0.304	1:100	0.316	1:100	(+)/(+)
8	M	0.168	No reagent	0.147	No reagent	(-)/(-)
9	M	0.116	No reagent	0.131	No reagent	(-)/(-)
10	M	0.401	1:200	0.382	1:200	(+)/(+)
12	M	0.251	1:100	0.283	1:100	(+)/(+)

**OD **- Optical Density. **ELISA - **Enzyme Linked immunosorbent assay. **Bac - **Baculovirus. ***E. coli *- ***Escherichia coli*. 1. Result of assay used IgM-ELISA-rN-Bac and IgM-ELISA-rN-*E. coli*.

**Table 4:** Evaluation of IgG ELISA-rN-Bac and IgG ELISA-rN-*E. coli *against sera from volunteers from the cities of Paraíso and Belmonte, SC.

*Patients/City and State of origin*	*Sex*	*Age*	*rN-Bac* *OD*	*rN-E. coli* *OD*	*Results^1^*	*Patients/City and State of origin*	*Sex*	*Age*	*rN-Bac* *OD*	*rN-E. coli* *OD*	*Results^1^*
1-Par-SC	M	20	0.109	0.139	(-)/(-)	26-Par-SC	M	24	0.219	0.240	(-)/(-)
2-Par-SC	F	31	0.167	0.141	(-)/(-)	27-Par-SC	M	69	0.782	0.992	(+)/(+)
3-Bel-SC	M	35	0.181	0.191	(-)/(-)	28-Par-SC	M	31	0.137	0.118	(-)/(-)
4-Bel-SC	F	50	1.058	0.912	(+)/(+)	29-Bel-SC	F	59	0.156	0.194	(-)/(-)
5-Par-SC	M	42	0.098	0.167	(-)/(-)	30-Bel-SC	M	30	0.194	0.173	(-)/(-)
6-Par-SC	M	86	1.218	1.054	(+)/(+)	31-Bel-SC	M	27	0.149	0.181	(-)/(-)
7-Par-SC	M	48	1.023	1.162	(+)/(+)	32-Par-SC	F	46	0.107	0.081	(-)/(-)
8-Par-SC	F	47	0.137	0.179	(-)/(-)	33-Bel-SC	F	18	0.864	0.719	(+)/(+)
9-Bel-SC	F	31	0.121	0.140	(-)/(-)	34-Par-SC	M	57	0.186	0.202	(-)/(-)
10-Bel-SC	F	37	0.129	0.104	(-)/(-)	35-Par-SC	M	40	0.243	0.217	(-)/(-)
11-Bel-SC	F	21	0.108	0.096	(-)/(-)	36-Par-SC	M	31	0.154	0.091	(-)/(-)
12-Par-SC	M	29	0.089	0.102	(-)/(-)	37-Par-SC	F	19	0.147	0.137	(-)/(-)
13-Par-SC	F	27	0.911	0.816	(+)/(+)	38-Par-SC	F	56	1.534	1.209	(+)/(+)
14-Bel-SC	M	45	0.097	0.089	(-)/(-)	39-Par-SC	F	26	0.136	0.143	(-)/(-)
15-Bel-SC	M	40	0.162	0.143	(-)/(-)	40-Bel-SC	M	62	0.098	0.079	(-)/(-)
16-Par-SC	F	39	0.141	0.156	(-)/(-)	41-Par-SC	F	76	0.942	0.985	(+)/(+)
17-Bel-SC	F	52	0.182	0.167	(-)/(-)	42-Par-SC	F	74	0.137	0.199	(-)/(-)
18-Bel-SC	M	56	1.102	0.977	(+)/(+)	43-Bel-SC	F	51	0.139	0.142	(-)/(-)
19-Par-SC	F	31	0.116	0.095	(-)/(-)	44-Bel-SC	F	27	1.367	1.418	(+)/(+)
20-Par-SC	F	60	0.087	0.073	(-)/(-)	45-Bel-SC	M	43	0.142	0.125	(-)/(-)
21-Par-SC	F	27	0.213	0.192	(-)/(-)	46-Bel-SC	M	36	0.101	0.089	(-)/(-)
22-Par-SC	M	39	0.183	0.207	(-)/(-)	47-Par-SC	F	31	0.128	0.151	(-)/(-)
23-Par-SC	F	57	1.006	1.355	(+)/(+)	48-Par-SC	M	20	0.191	0.168	(-)/(-)
24-Bel-SC	M	24	0.173	0.197	(-)/(-)	49-Par-SC	M	48	0.639	0.567	(+)/(+)
25-Par-SC	M	37	0.094	0.056	(-)/(-)	50-Bel-SC	F	38	0.208	0.231	(-)/(-)

**Par- **Paraíso. **Bel**- Belmonte. **SC - **Santa Catarina. **OD **- Optical Density. **ELISA - **Enzyme Linked immunosorbent assay. **Bac - **Baculovirus. ***E. coli *- ***Escherichia coli*. 1. Results of IgG-ELISA-rN-Bac and IgG-ELISA-rN-*E. coli*.

## Discussion

The production of native antigens for Hantavirus serological tests has several limitations, including a high risk of contamination by handling the virus, low viral titers obtained in cell culture, difficulty in adapting the virus to cells, slow viral replication (3 to 10 days), and occurrence of virus mutations after successive passages in cell culture [15]. Thus, Hantavirus antigens produced by recombinant DNA technology became a common approach for this purpose [16]. The N protein contains important viral antigens and has been previously produced by recombinant techniques for use in serologic diagnosis [17,18]. In the present study, we have successfully expressed the recombinant N protein of Araraquara Hantavirus in insect cells by recombinant Baculovirus expression system. Since the N gene was under control of very late baculovirus promoter, high levels of the rN-Bac were obtained after 72 h p.i. as observed by other authors expressing their proteins in insect cells using similar vectors [15,19-22]. However, rN-Bac was mostly present in intra-cytoplasmatic inclusion bodies and required denaturing buffer solutions for a adequate purification. The recombinant protein was analyzed by western blot showing suitable antigenic characteristics by strongly reacting with human sera from HCPS patients.

The rN-Bac was used as an antigen in an IgG and IgM ELISA which was able to detect anti-nucleoprotein antibodies in both human and rodent sera (Data not shown). This Hantavirus rN-Bac IgG-ELISA was compared to a similar test using N recombinant protein antigen produced in *E. coli*. The rN-*E. coli *is used in Brazil as the regular antigen for ELISA diagnosis of Hantavirus infections. Eighty human serum samples were tested by both ELISAs showing equal results, 23 samples were positive and 67 were negative. No significant differences were observed for optical density values and test cut-off values in both tests. Hantavirus rN-Bac and rN-*E. coli *IgM ELISAs showed both to be usefull as diagnostic tools. Among eleven tested sera, 6 were positives and 5 negatives in both tests. Thus, no significant differences were observed on optical densities, serum titers and the cut-offs of both tests showed close values. These results showed that the IgG or IgM ELISA using rN-Bac as antigen were equally effective to the rN-*E. coli *IgG or IgM ELISA in terms of sensitivity and specificity.

Previous comparison of serological methods for the diagnosis of Hantavirus infection indicated that ELISA assays with N protein produced in *E. coli *was less sensitive than assays performed with N protein produced in insect cells through the Baculovirus system [11]. The same study also showed that even after several rounds of purification the presence of residues from *E. coli *could still generate false positive results [11].

## Conclusion

An N recombinant protein of Araraquara Hantavirus was successfully produced in insect cells and the preliminary tests of this protein as an ELISA antigen encourage its use for diagnosis of HCPS and seroepidemiological surveys of Hantavirus in Brazil.

## Competing interests

The authors declare that they have no competing interests.

## Authors' contributions

**AMM **held the cloning and expression of recombinant N protein of Hantavirus Araraquara. Participated in the tests of ELISA using antigen produced in both baculovirus and *E. coli*. Also, participated in data analysis and drafted the manuscript. **ARSRM **participated in both ELISA and data analysis. **MLM **provided the Araraquara virus N protein expressed in *E. coli*, and performed the standardization of the ELISA using this antigen. **BMR **provided the expression vector (pSynXIVVI+X3), Sf9 cells, baculovirus vSynVI^-^gal. Participated in the production of recombinant baculovirus. **LTMF **provided the Araraquara virus N protein expressed in *E. coli*, and performed the standardization of the ELISA using this antigen. Conceived of the study, and participated in its design and coordination and helped to draft the manuscript. **JLCW **conceived the study, participated in its design and coordination, data analysis and helped to draft the manuscript. All authors read and approved the final manuscript.
